# Editorial: Harnessing the Power of Patient Derived Models of Cancer

**DOI:** 10.3389/fonc.2018.00349

**Published:** 2018-09-06

**Authors:** Sharon R. Pine, Hatem E. Sabaawy

**Affiliations:** ^1^Department of Medicine, Robert Wood Johnson Medical School, Rutgers, The State University of New Jersey, New Brunswick, NJ, United States; ^2^Department of Pharmacology, Robert Wood Johnson Medical School, Rutgers, The State University of New Jersey, New Brunswick, NJ, United States; ^3^Rutgers Cancer Institute of New Jersey, Rutgers, The State University of New Jersey, New Brunswick, NJ, United States

**Keywords:** PDX (patient derived xenograft), PDOX (patient-derived orthotopic xenograft), tumor heterogeneity, precision medicine, preclinical models of cancer

We are honored to introduce a collection of five original research articles, protocols, reviews, and perspectives on the research topic “Harnessing the Power of Patient Derived Models of Cancer.” Gone are the days when we rely solely on decades-old cancer cell line cultures in the quest for determining and predicting therapeutic responses. Preclinical studies now rely heavily on patient-derived cancer models to guide the development and implementation of targeted therapies, leading to major improvements in drug validation and discovery platforms, and ultimately improving patient survival and quality of life.

Exploration of cancer “omics” has been increasing exponentially. Parallel to the omics revolution has been the rising use of early passage patient-derived tumor cell cultures (two- and three-dimensional) (2D and 3D), in particular in sphere and organoid 3D models, and/or patient-derived xenograft (PDX) models. The technologies for deriving many of these models have been explored for half a century, but their wide-spread implementation has surged with our deepened appreciation for tumor heterogeneity, tumor cell clonal evolution, and the importance of cell-cell and cell-extracellular matrix interactions within the tumor microenvironment. Patient-derived cancer models provide an improved platform to study cancer biology and a more representative delineation of the therapeutic response. The models facilitate the integration of omics data with drug sensitivity data on a personalized basis and currently represent the ultimate approach for preclinical drug development and biomarker discovery.

In this issue, Morgan et al. explore the recent advances in subcutaneous PDX mouse models of non-small cell lung cancer (NSCLC). The benefits of NSCLC PDX mouse models and their ability to reflect the parental tumors' histomorphological characteristics are deliberated. Comparisons between clonal selection and evolution in low-passage PDXs are also highlighted and further related to the donor tissue. The authors raise vital questions regarding the practical utility of PDXs and the use of humanized PDX models in predicting patient response to therapy and also provide recommendations for addressing those critical questions.

Other tumor types such as brain tumors are better suited for patient-derived orthotopic xenograft (PDOX) mouse models, because they preserve the local tumor microenvironment that is necessary for optimal drug discovery (Patrizii et al.). The authors present the key advantages, disadvantages, and potential applications of cell- and animal-based models of glioblastoma (GBM). They highlight their experience with the GBM 3D culture of patient-derived tumor stem-like cells in sphere and organoid cultures and interchangeably in the mouse PDOX model. The authors highlight the utility of these models in drug discovery, particularly for identifying effective compounds that also could essentially pass the blood-brain barrier. They discuss the factors that define the self-renewal potential of GBM stem-like cells, and demonstrate the ability to generate clonal patient derived 3D cultures to represent the variety of heterogenous clones within patient samples. PDOXs derived from GBM stem-like cells recapitulated key GBM morphological, architectural and expression feature that are present in primary GBM. Moreover, using stem-like cells to initiate 3D cultures could shorten the overall time for model generation, and allow a focus on cells that are resistant to conventional therapy, thus reflecting a benefit for developing better and more effective targeted and personalized therapies for GBM patients.

You cannot mention advances in patient-derived cancer models without discussing the importance of intra-tumor heterogeneity. Potent and lasting therapy requires a deep understanding of intra-tumoral clonal evolution, cell plasticity, and interactions between tumor cells and their microenvironment. Gambara et al. describe how *in vitro* and *in vivo* cancer models resembling the genomic and phenotypic heterogeneity of patient tumors are required for tailoring treatments to the individual composition of the tumor. The authors discuss tumor cellular hierarchy and factors contributing to intra-tumor heterogeneity. They highlight the suitability of 3D models for multi-regional and longitudinal tumor sampling, studying immune-tumor cell interplay, and use for genome editing, high-throughput screening, and personalized therapy. They highlight the need for coalescence of 3D and PDX models for a better understanding of tumor evolution and immune interventions.

Laboratories world-wide have been touting their ability to generate patient-derived cancer models. Generation of a few PDXs is relatively easy to achieve, but the requirements for building PDX and organoid tumor banks to represent heterogenous tumors can be daunting. The acquisition, propagation, annotation and distribution of patient-derived tumor samples are complex and costly. It requires a high degree of coordination among clinical, surgical, regulatory, and laboratory personnel, and is fraught with challenges that are administrative, procedural and technical. Mattar et al. examine the major aspects of this complex process and relate their experience in maintaining a PDX Core facility within a large academic institution. They provide helpful workflow, operating procedures and quality control considerations for seamless generation of a large number of “off-the-shelf” and well-characterized PDXs for use in preclinical studies.

A major purpose of generating patient-derived cancer models for preclinical studies is to learn how to tailor therapies to the unique profiles of the individual patient tumors. To effectively reach this goal, we need to generate models from patients with a wide range of racial, cultural, and ethnic backgrounds. We are learning that patients from different countries are more or less inclined to consent to their tissues to be used in research studies. Labib et al. describe the factors affecting whether parents of pediatric cancer patients are willing to donate their children's samples for research and tissue banking. The authors found that older and more highly educated parents may require different communication methods. Their study highlights the need for interviewers to present the research study in various ways according to the level of participants' understanding.

We welcome you to use this issue as a reference. As with “omics” data, patient-derived cancer models have advanced and their use is becoming a mainstay. Surely, these models are improving our ability to understand tumor heterogeneity, develop, and test anti-cancer agents and tailor therapies to patients, leading to ultimately improved survival and better quality of life.

## Author contributions

All authors listed have made a substantial, direct and intellectual contribution to the work, and approved it for publication.

### Conflict of interest statement

The authors declare that the research was conducted in the absence of any commercial or financial relationships that could be construed as a potential conflict of interest.

